# Tightened social distancing measures and increased violence during the COVID-19 pandemic in South Korea

**DOI:** 10.3389/fpsyg.2023.1152693

**Published:** 2023-07-04

**Authors:** Bookyoung Kim, Kyung-Bok Son

**Affiliations:** ^1^Seoul Hongyeon Elementary School, Seoul, Republic of Korea; ^2^College of Education, Hanyang University, Seoul, Republic of Korea; ^3^College of Pharmacy, Hanyang University, Ansan, Republic of Korea

**Keywords:** social distancing, COVID-19, violence, hot-lines, South Korea

## Abstract

**Introduction:**

In 2020, the South Korean government introduced social distancing measures, varied by region, to address the pandemic. We captured variations in social distancing measures among regions in South Korea and investigated the association between the stringency of measures and the increased incidence of violence.

**Methods:**

Incidence reports from calls to violence hotlines, including school and domestic violence and sexual harassment, from 2016 to 2021 were retrieved. The regional *per capita* incidence rates for each violence hotlines were calculated. Difference-in-difference design with fixed effects was used to elucidate different trends in the incidence rate of violence between regions with stringent social distancing measures and regions with looser measures.

**Results:**

Social distancing measures led to a decreased incidence rate of school violence and an increased incidence rate of domestic violence and sexual harassment. Different trends in the incidence of violence were noted between regions with strict social distancing measures and regions with more lenient measures. Tightened measures caused surges in domestic violence and sexual harassment.

**Conclusion:**

Social distancing measures have been an inevitable mitigation strategy against virus transmission throughout the pandemic. However, women residing in tightened social distancing measures, in particular urban areas, need more support against domestic violence.

## Introduction

The COVID-19 pandemic has tremendously impacted public health (Hartley and Perencevich, 2020; Heymann and Shindo, 2020). Governments have devised various measures to control the spread of the virus (Ayouni et al., 2021). Government measures include social and physical distancing restrictions (Venkatesh and Edirappuli, 2020), mandated stay-at-home and business closure rules (Bendavid et al., 2021), the implementation of online school and remote work practices (Suryaman et al., 2020), and lockdowns (Sameer et al., 2020). These measures, compounded by fear and the stigma of being infected, have caused radical shifts in everyday life (Bagcchi, 2020). One such modification has dramatically reduced activity and mobility (Borkowski et al., 2021), implying increased time spent at home with family members.

Measures during the pandemic will be linked to far-reaching social, financial, and psychological consequences (Boserup et al., 2020). The economic effects of the pandemic seem to have been linked to the increased incidence of violence (Bitler et al., 2020). The pandemic has created a default context for families living at home under chronic economic uncertainty associated with high stress, anxiety, and irritation (Brooks et al., 2020). Staying at home with perpetrators during the pandemic may have exacerbated vulnerabilities in victims of violence (Anurudran et al., 2020; Bradbury-Jones and Isham, 2020; Piquero et al., 2021). Increased domestic violence and child abuse may be associated with social and physical distancing measures under which family members are confined to their homes without access to those who may notice the signs of violence and/or assistance necessary for victims to escape the violence (Leslie and Wilson, 2020).

### Brief sketch of the COVID-19 pandemic in South Korea

In South Korea, a passenger arriving at Incheon International Airport, Incheon from Wuhan, China, was detected as the first confirmed case of COVID-19 on January 20, 2020 (Son et al., 2021). The number of confirmed cases slightly increased within a month after detecting the first case. Three discrete peaks occurred in February, August, and November 2020 (Son, 2022). Supplementary Table S1 shows the social distancing plan proposed by the government to address the pandemic. The government implemented “enhanced distancing” to regulate the operation of multi-person facilities on March 22, 2020. The mayor of Seoul issued an administrative order to ban gatherings in pubs, bars, and karaoke clubs on April 8, 2020, and he relaxed the order on April 20, 2020. On June 28, 2020, the measures were officially re-named “social distancing,” and tactics have transformed several times according to the severity of the pandemic. The number of confirmed cases rapidly increased in the metropolitan area, including Seoul, Incheon, and Gyeonggi regions, since August 2020. The government introduced social distancing measures, varied by region, to address outbreaks concentrated in the metropolitan area. Level 2.5 was effective in the metropolitan area, while level 2 was in effect in the non-metropolitan area (Dighe et al., 2020).

As one of the consequences of differential social distancing tactics, Supplementary Table S2A shows the operation plan for school in the first semester of 2020. The government adopted a country-wide school operation plan. In 2020, the first day of the new school year was scheduled to fall on March 1. Instead, the Ministry of Education postponed the day to March 23 and April 6. Finally, phased-in online schooling was introduced to compensate for the increased risk of group transmission among students. On April 9, students in grades 9 and 12 started online schooling, followed by students in grades 4–8, 10, and 11 on April 16, and students in grades 1–3 on April 20. The government separated the school operation plan into metropolitan and non-metropolitan areas according to the severity of the pandemic in the second semester of 2020. Supplementary Table S2B describes the split plans for schools in two areas. The numbers in the table indicate the ratio of students who could attend school in person. For instance, one-third of elementary and middle school students in the metropolitan area could attend school in person after August 16. However, attending school was stopped for grades 1–11 students on August 30 in the metropolitan areas. In contrast, schools in non-metropolitan areas adopted relatively loosened measures.

This study analyzed the effects of social distancing measures on the incidence of violence in various forms. The effectiveness of social distancing measures in mitigating the spread of the virus has been reported (Qian and Jiang, 2020; Thu et al., 2020). The literature has also demonstrated the effects of social distancing measures on violence in various forms (Goh et al., 2020; Hsu and Henke, 2021). However, evidence of the association between the stringency of social distancing measures and the incidence of violence is scarce. We captured variations in social distancing measures among regions in South Korea and investigated the association between the degree of measures and the increased incidence of violence. Metropolitan areas maintained tightened measures to address ongoing surges in confirmed cases, whereas the remaining regions adopted relatively loosened measures. We used difference-in-difference (DID) methods with fixed effects to compare the incident rate of violence between regions with tightened and loosened measures. Findings from this study may shed light on establishing adequate social distancing measures in terms of effectiveness in mitigating virus transmission and protecting the population from violence.

## Methods

### Data source

Two datasets were obtained to calculate a regional *per capita* incidence rate of school and domestic violence and sexual harassment. First, incidence reports from calls to hotline centers from 2016 to 2021 were retrieved (National Policy Agency, 2020). The National Policy Agency (NPA) operates 117 hotline centers to support victims of school, domestic, and sexual violence. The centers provide emergency rescue, police investigation, and legal advice for victims. Victims can also report violence through phone calls, text messages, and/or visits to centers located in the 16 regions (Policy Agency for Children, W., and Disabled, 2022). The 16 regions are categorized into *Si* and *Do. Si* indicates urban areas and includes 7 regions of Seoul, Busan, Daegu, Incheon, Gwangju, Daejeon, and Ulsan. *Do* indicates rural areas and includes 9 regions of Gyeonggi, Gangwon, Chungbuk, Chungnam, Jeonbuk, Jeonnam, Gyeongbuk, Gyeongnam, and Jeju (National Policy Agency, 2020). Second, registry data provided by the Ministry of the Interior and Safety was used to calculate a regional *per capita* incidence rate. The number of populations 6–17 years old was retrieved to calculate a regional *per capita* incidence rate of school violence. In a similar vein, the number of the female population 20–65 years old was retrieved to calculate a regional *per capita* incidence rate of domestic violence and sexual harassment.

### Measures

#### Dependent variable

The dependent variable for this study is the regional *per capita* incidence rate of school (per 100,000 students 6–17 years old) and domestic violence and sexual harassment (per 100,000 females 20–65 years old) from 2016 to 2021 in 16 South Korean regions.

#### Independent variables

The association between the stringency of social distancing measures and violence is not clear. However, tightened measures would cause increased violence (Kourti et al., 2023). This study captures variations in social distancing measures among regions and investigates the association between social distancing measures and trends in the *per capita* incidence rate of violence. During the initial stages of the pandemic, the number of confirmed COVID-19 cases surged in metropolitan areas. The metropolitan area promptly adopted strict measures to contain the spread of the virus because approximately 50% of South Korea’s total population is concentrated in this area (12% of the total geographical area). For purposes of analysis, we treated the regions in metropolitan areas as treatment groups and the remaining regions as control groups. Note that 2 *Si*s out of 7 and 1 *Do* out of 9 were assigned to the treatment group. The remaining 5 *Si*s and 8 *Do*s were assigned to the control group. Such a study design should rest on the assumption that each area’s assignment to treatment or control groups is as good as a random assignment.

#### Statistical analysis

This study applied two types of analysis: descriptive and DID analysis. First, we presented the *per capita* incidence rate of school violence, domestic violence, and sexual harassment from 2016 to 2021 in national, urban, rural, metropolitan, and non-metropolitan areas. Second, we used the DID method to elucidate differences in trends between regions with strict social distancing measures and regions with relatively loosened measures. Unobserved variables that systematically vary across regions and years might affect a regional incidence rate. The DID method with fixed effects reduce the risk of time-invariant confounding factors (Vecino-Ortiz and Guzman-Tordecilla, 2020). We used fixed effects to remove unobserved heterogeneity between regions and years in our datasets. Finally, we conducted the following models with heteroscedasticity consistent standard errors as the main analysis.


$$y_{it}=\beta_{0}+\beta_{1}\;\left(Treat_{i}\right)+\beta_{2}\;\left(Post_{t}\right)+\beta_{3}\;\left(Treat_{i}\;X\;Post_{t}\right)+\gamma C_{it}+\varepsilon_{it}$$

*Treat* is a dummy variable for each region that equals 1 if the region is in the treatment group (i.e., metropolitan areas). *Post* is a dummy variable for each year that equals 1 if the year is 2020 or 2021. *C* is a set of region and year dummies. Metropolitan areas, which include two *Si*s and one *Do*, were assigned to the treatment group; the remaining areas, which include five *Si*s and eight *Do*s, were assigned to the control group. For the coefficient, β_0_ is the baseline average; β_1_ is the difference between the two groups pre-intervention; β_2_ is the time trend in the control group; and β_3_ is the difference in changes over time. We conducted the DID model separated in urban and rural areas for the sub-group analyses. Data management and analysis were performed using R statistical software (version 4.1.2). Statistical significance was noted when *p*-values were less than 0.05.

## Results

### Characteristics of two areas

Table 1 presents the characteristics of two areas in South Korea. The metropolitan area, including 2 *Si*s and 1 *Do*, comprises 50% (26 million) of the total population and 12% (11 thousand km^2^) of the whole area, indicating that the population density in this area is much higher than that of the remaining area (2,188 persons/km^2^ versus 292 persons/km^2^). COVID-19 cases per area were more elevated in the metropolitan area than in the non-metropolitan area. The accumulated COVID-19 cases in the metropolitan area were 33,754 on December 31, 2020, resulting in 2.8 cases per 1 km^2^ and 1.3 cases per 1,000 persons, respectively. In contrast, the number of COVID-19 cases in the non-metropolitan area, including 5 *Si*s and 8 *Do*s, was 20,661 on December 31, 2020, resulting in 0.2 cases per 1 km^2^ and 0.8 cases per 1,000 persons, respectively.

**Table 1:** Basic characteristics and COVID-19 cases in metropolitan and non-metropolitan areas of South Korea.

Basic characteristics	Metropolitan (2 *Si*s and 1 *Do*)			Non-metropolitan (5 *Si*s and 8 *Do*s)		
Region	Seoul, Incheon, and Gyeonggi			The remaining region		
Land area (km2)	11,865 (12%)			88,548 (88%)		
Population (1,000 persons)	25,958 (50%)			25,823 (50%)		
Population density (persons/km^2^)	2,188			292		
Health care institutions (excluding pharmacies)	34,882 (52%)			32,218 (48%)		
Hospital beds	252,296 (36%)			444,662 (64%)		
COVID-19 cases	Cases	Per km^2^	Per 1,000 persons	Cases	Per km^2^	Per 1,000 persons
2020 1Q	990	0.083	0.038	8,796	0.099	0.341
2020 2Q	2,860	0.241	0.110	9,940	0.112	0.385
2020 3Q	9,496	0.800	0.366	11,088	0.125	0.429
2020 4Q	33,754	2.845	1.300	20,661	0.233	0.800

### Trends in reported violence from 2016 to 2021

Table 2 presents the *per capita* incidence rate of school violence, domestic violence, and sexual harassment from 2016–2021 in national, urban, rural, metropolitan, and non-metropolitan areas. At the national level, the *per capita* incidence rate on school violence hotlines increased in 2017, decreased until 2019, plummeted in 2020, and increased in 2021. The *per capita* incidence rate on domestic violence hotlines presented decreased trends until 2019 and increased in 2020. The *per capita* incidence rate on sexual harassment hotlines fluctuated until 2019 and surged in 2020.

**Table 2:** *Per capita* incidence rate of school and domestic violence and sexual harassment in national, urban, rural, metropolitan, and non-metropolitan region.

	National (7 *Si*s and 9 *Do*s)	Urban (7 *Si*s)	Rural (9 *Do*s)	Metropolitan (2 *Si*s and 1 *Do*)	Non-metropolitan (5 *Si*s and 8 *Do*s)
School violence: mean (standard deviation)					
2016	1068.03 (214.69)	1201.37 (164.71)	964.31 (195.99)	1176.02 (255.21)	1043.21 (207.77)
2017	1176.81 (259.39)	1355.01 (209.27)	1038.20 (209.55)	1301.41 (299.79)	1148.05 (253.67)
2018	1048.34 (247.76)	1205.31 (184.38)	926.26 (226.44)	1131.46 (211.61)	1029.16 (259.12)
2019	1021.04 (258.35)	1151.80 (208.37)	919.33 (256.87)	1128.54 (204.06)	996.23 (270.06)
2020	524.59 (128.87)	561.61 (123.92)	495.80 (132.25)	461.44 (157.55)	539.17 (124.08)
2021	712.18 (133.37)	745.89 (129.69)	685.95 (137.73)	639.18 (131.76)	729.02 (133.05)
Domestic violence: mean (standard deviation)					
2016	7.56 (3.45)	8.89 (4.47)	6.54 (2.14)	9.97 (7.15)	7.01 (2.14)
2017	5.20 (3.76)	6.87 (5.14)	3.91 (1.53)	8.64 (7.72)	4.41 (2.02)
2018	5.12 (4.98)	7.14 (7.16)	3.55 (1.31)	9.89 (11.29)	4.02 (1.66)
2019	4.65 (3.92)	6.13 (5.62)	3.51 (1.31)	9.18 (7.83)	3.61 (1.64)
2020	4.88 (6.84)	6.67 (10.42)	3.49 (1.21)	12.44 (15.10)	3.13 (1.73)
2021	5.51 (6.46)	7.51 (9.57)	3.96 (1.85)	12.56 (12.80)	3.88 (3.09)
Sexual harassment: mean (standard deviation)					
2016	2.41 (1.69)	2.18 (1.67)	2.58 (1.78)	2.79 (2.50)	2.32 (1.57)
2017	1.67 (1.78)	1.70 (2.61)	1.64 (0.90)	3.06 (3.78)	1.35 (0.98)
2018	2.12 (3.26)	3.11 (4.61)	1.35 (1.57)	4.90 (7.26)	1.48 (1.45)
2019	1.86 (3.85)	3.01 (5.82)	0.97 (0.59)	5.72 (9.07)	0.97 (0.48)
2020	3.20 (6.16)	5.63 (4.70)	1.31 (0.61)	9.61 (14.10)	1.72 (1.32)
2021	2.82 (6.83)	4.70 (10.41)	1.36 (0.83)	9.85 (15.92)	1.20 (0.93)

Table 2 also presents the difference in the incidence rate between 2019 and 2020, so-called before and after the pandemic. The *per capita* incidence rate of school violence decreased by 496 (49%). The increase in the *per capita* incidence rate of domestic violence and sexual harassment was 0.2 (5%) and 1.3 (72%), respectively. In urban and rural areas, the *per capita* incidence rate of school violence decreased by 590 (51%) and 424 (46%), respectively. The *per capita* incidence rate for domestic violence increased by 0.5 (9%) in urban areas. However, the value was marginally decreased by 0.02 (−1%) in rural areas. The *per capita* incidence rate of sexual harassment increased by 2.6 (87%) and 0.3 (35%) in urban and rural areas, respectively. In metropolitan and non-metropolitan areas, the *per capita* incidence rate of school violence decreased by 667 (59%) and 457 (46%), respectively. The *per capita* incidence rate for domestic violence increased by 3.3 (36%) in urban areas. However, the value was decreased by 0.5 (−13%) in rural areas. The *per capita* incidence rate of sexual harassment increased by 3.9 (68%) and 0.8 (77%) in urban and rural areas, respectively.

### Difference-in-difference design with fixed effects

Table 3 presents the results of DID model with fixed effects. We found differences in trends between regions with strict social distancing measures and regions with more lenient measures. The *per capita* incidents reported to school violence hotlines (−214.01) decreased in the regions with tightened social distancing measures. The *per capita* domestic violence incidents (4.33) and sexual harassment (5.68) increased in the regions with strict social distancing measures. In the sub-group analysis, we found that the effect of stringency of social distancing measures on school violence was more remarkable in rural (−232.84) than in urban areas (−122.34). The effect of stringency on domestic violence and sexual harassment were significant in urban areas (7.47 and 7.70). However, the effects were insignificant in rural areas (−0.48 and 0.89).

**Table 3:** Results of difference-in-difference with fixed effects regression.

	School violence			Domestic violence			Sexual harassment		
	Estimate	Standard Error	P-value	Estimate	Standard Error	P-value	Estimate	Standard Error	P-value
National (7 *Si*s and 9 *Do*s)									
Treated	169.01	58.19	0.0048	17.76	1.57	<0.0001	13.72	2.52	<0.0001
Post	−315.72	37.19	<0.0001	−2.86	0.67	<0.0001	−0.65	0.68	0.3467
DID	−214.00	38.14	<0.0001	4.33	1.51	0.0053	5.68	2.35	0.0184
Urban (7 *Si*s)									
Treated	138.45	57.06	0.0217	16.72	1.34	<0.0001	13.04	2.19	<0.0001
Post	−420.52	47.50	<0.0001	−3.51	1.32	0.0127	0.31	1.29	0.8090
DID	−122.34	46.96	0.0143	7.47	1.93	0.0005	7.70	3.14	0.0206
Rural (9 *Do*s)									
Treated	−20.46	48.43	0.6749	−0.55	0.38	0.1580	−1.12	0.26	0.0001
Post	−252.48	45.68	<0.0001	−2.52	0.54	<0.0001	−1.32	0.46	0.0073
DID	−232.84	46.35	<0.0001	−0.49	0.40	0.2250	0.89	0.37	0.2181

## Discussion

Throughout the COVID-19 pandemic, governments have devised various measures to mitigate the spread of the virus. Social distancing and staying at home during times of high uncertainty and stress may have exacerbated threatening situations for victims of violence. The literature has found that while social distancing measures have effectively mitigated the spread of the virus (Goh et al., 2020; Hsu and Henke, 2021), social distancing has also exacerbated violence (Qian and Jiang, 2020; Thu et al., 2020). However, evidence on the associations between social distancing measures and the incidence of violence is scarce. This study elucidates the effects of the stringency of measures on the incidence of violence in various forms.

### Violence in schools and at home during the pandemic

During the pandemic, increased domestic violence was reported in the United States, the United Kingdom, and Australia (Leslie and Wilson, 2020; Matthew, 2020; Neil, 2020; Piquero et al., 2021). In the United States cities, the pandemic led to a 7.5% increase in calls for service for domestic violence during the initial stage of COVID-19 (Leslie and Wilson, 2020). South Korea is no exception. The *per capita* incidence rate of domestic violence and sexual harassment increased, while the *per capita* incidence rate of school violence plummeted right after the pandemic. This study found that stricter measures caused surges in domestic violence and sexual harassment and decreased school violence. These findings are well explained by the routine activity theory, a sub-field of crime opportunity theory that focuses on crime situations. The theory provides a macro perspective on crime and describes how changes in social and physical environments impact crimes. Under this approach, a motivated offender must physically contact a suitable target without capable guardians who make committing the crime more difficult (Cohen and Felson, 1979). The pandemic has changed the relationship among offenders, targets, and guardians (Eck and Madensen, 2015). Under social distancing measures, the offender and the target of domestic violence and sexual harassment are more likely to be in the same place without a guardian. With school violence, in contrast, the offender and the target were less likely to be in the same place under the circumstances of online schooling. The phase-in model of online schooling decreased the chances of physical contact among students, contributing to reduced school violence.

### Trends in domestic violence in South Korea

This study investigated domestic violence before and after the pandemic in urban, rural, metropolitan, and non-metropolitan regions. Several findings are noteworthy.

First, before the pandemic, the *per capita* incidence rate of domestic violence had decreased. Domestic violence arises within social contexts (Jewkes, 2002). Under the influence of the Confucian culture, domestic violence had been assumed to be tolerated to maintain family structure (Shim and Nelson-Becker, 2009). Women in South Korea were silent and obedient regarding domestic violence. In the 1990s, a tacit consensus prevailed that domestic violence was a private matter (Kim, 1998). However, domestic violence has gained policy attention with growing concerns about the victimization of women and the changed role of sex (Lee, 2008). The spread of severe domestic violence cases through mass media prompted the government to make legislation.

In July 1998, the government implemented the Prevention of Domestic Violence and Victim Protection Act and the Special Act for the Punishment of Domestic Violence (Kim et al., 2016). Following the legislation, domestic violence in South Korea continuously decreased in the 2010s (Kim et al., 2016). However, the prevalence was still higher than in other countries. Decreased trends in the rate of domestic violence from 2016 to 2019, reported in this study, could be understood in this context. Furthermore, we found that the standard deviation of domestic violence had increased during the observation period. This observation implies that decreased domestic violence would be associated with regional factors.

Second, the rate of domestic violence was higher in urban than rural areas. Similarly, the rate of domestic violence was more elevated in metropolitan than in non-metropolitan areas. In contrast, it was reported that the rates of domestic violence were similar across urban and rural areas (Edwards, 2015). A higher prevalence of domestic violence and severe forms of physical abuse were reported for women in rural areas than for urban women (Peek-Asa et al., 2011).

Socio-demographic factors, including age, education, employment, and income, are associated with domestic violence. Low socio-demographic characteristics were significant risk factors for physical violence by men (Kim et al., 2016). In this vein, social disorganization theory suggests that crime rates, including domestic violence, would be higher in areas with high poverty levels (Kubrin and Weitzer, 2003). Fewer community resources, geographic and social isolation, and patriarchal family structure would be linked to the increased prevalence of domestic violence. Thus, domestic violence in rural or non-metropolitan areas would be more elevated than in urban or metropolitan areas. However, we found contrasting trends in domestic violence in South Korea. Increased rates of domestic violence in urban or metropolitan areas might be associated with the self-role discrepancy theory (Yang et al., 2018). The theory suggests that those who violate socialized sex roles suffer adverse psychological consequences. Men with low socio-demographic status in urban areas would experience psychological difficulties such as an inferiority complex.

Third, not surprisingly, social distancing measures have a detrimental effect on the prevalence of domestic violence. However, the degree of effect was varied by the strength of social distancing measures and/or the region. In the DID model, the effect of social distancing measures on domestic violence was higher in areas with tighter measures. In the sub-group analysis, we found that the effect was higher in urban than rural areas. These findings imply that tightened social distancing measures had a more negative impact on women in urban areas.

### Policy measures to address violence

Inequalities in social determinants of health are magnified during a pandemic (Evans et al., 2020). The pandemic has caused increased domestic violence because people are confined to their homes. Staying at home does not provide equivalent safety for all people. Based on the experience in South Korea, we suggest policy options to address surged violence during the pandemic.

The role of an observer or one who finds hidden violence should be emphasized as opposed to that of a guardian who makes committing the violence difficult. In the pandemic, medical professionals have opportunities to find signs of hidden violence in patients in healthcare settings and connect victims with social services. Telemedicine and mobile health have the potential to contribute to finding invisible victims who are not able to visit health centers (Roesch et al., 2020). In a similar vein, teachers have the potential to capture violence at in-person or online schools. Medical professionals and teachers should be aware of the risks of violence against women and children. Furthermore, providing adequate assistance to escape situations of violence should be guaranteed despite their limited operations during the pandemic. Domestic violence increased, but most institutions providing shelter and services for victims were closed during the pandemic. Under systemic disruption to social services, the victims reporting violence were more likely to be exposed to serious and possibly repeated violence without adequate protection. Social institutions to protect the victims should be prioritized in terms of funding and staffing to guarantee essential services for victims.

During the pandemic, violence could come in different ways, including emotional, psychological, physical, economic, and sexual forms (Pereda and Díaz-Faes, 2020; UN Women, 2020; Fawole et al., 2021). Cyberbullying and verbal forms of violence at school have continuously increased during the pandemic in South Korea (Ministry of Education, 2021). Likewise, domestic violence in psychological form has increased even while incidents of domestic violence in physical form have decreased in Latin America (Carreras and Perez-Vincent, 2021). However, these new forms of violence are not easily captured under the current reporting system. Thus, effective violence reporting mechanisms that capture various forms of violence should be established. Research proves that hotline channels better respond to victims’ needs than emergency lines and policy complaints (Carreras and Perez-Vincent, 2021). Mobile applications may be another option to identify victims of violence in this circumstance, where 93% of adults in South Korea had and used a smartphone in 2020 (Gallup, 2020). The government has developed the application so-called “Student Health Conditions Self-Diagnosis” to assess the health conditions of students (The Ministry of Education, 2022). On a daily basis, students or their parents must answer a 5-item questionnaire for physical attendance at school. The questionnaire includes items to assess physical symptoms suspected to be those of COVID-19 and the travel history of students and their families. A questionnaire item regarding violence in various forms could be included in the application to identify hidden victims.

### Study limitations

This study has several limitations. First, we collected reported violence from calls to hotlines across South Korea. However, reported violence might significantly underestimate the real incidence of violence. Similarly, reporting rates might be different across the country. Second, we used aggregated regional data to understand violence trends in the early stages of the pandemic. This study did not consider characteristics of victims and violence, such as the demographics of people calling violence hotlines, due to the lack of relevant data. Third, South Korea successfully controlled the spread of the virus in the initial stages of the pandemic. Nevertheless, the number of confirmed cases has surged again since March 2022. The number of confirmed cases was 353,980 on March 23, 2022, the highest number of daily confirmed cases recorded in any country. The long-term effects of the pandemic on violence in various forms will depend on the interaction between the intensity and the duration of the pandemic. Thus, findings from this study are limited to the initial stages of the pandemic and cannot be generalized to other periods of the pandemic. Finally, we adopted fixed effects to remove unobserved heterogeneity across regions and years. However, time-varying factors could cause bias in the estimation.

## Conclusion

Social distancing measures have been an inevitable mitigation strategy against virus transmission throughout the pandemic. The COVID-19 pandemic and governmental measures to contain the virus have caused far-reaching consequences for segments of the population. Policymakers should acknowledge that the strength of social distancing measures is associated with the increased incidence of violence. The government should consider the stringent social distancing measures against the costs of increased violence and the benefits of containing the virus. Women residing in tightened social distancing measures, in particular urban regions, need more support against domestic violence. The ongoing epidemic of violence should be prioritized as a public health problem to be addressed in conjunction with bringing the pandemic crisis to a close.

## Data availability statement

The data that support the findings of this study are available in Statistical Report provided by the National Policy Agency. These data were derived from the following resources available in the public domain: https://www.police.go.kr/.

## Author contributions

K-BS and BK conceived and designed the analysis, performed the analysis, and wrote the manuscript. All authors contributed to the article and approved the final version.

## Funding

This work was supported by the National Research Foundation of Korea (NRF-2022R1F1A1071338). However, the funder was not involved in the study design, collection, analysis, interpretation of data, the writing of this article or the decision to submit it for publication.

## Conflict of interest

The authors declare that the research was conducted in the absence of any commercial or financial relationships that could be construed as a potential conflict of interest.

## Publisher’s note

All claims expressed in this article are solely those of the authors and do not necessarily represent those of their affiliated organizations, or those of the publisher, the editors and the reviewers. Any product that may be evaluated in this article, or claim that may be made by its manufacturer, is not guaranteed or endorsed by the publisher.
