# Supplementary material for: Tightened social distancing measures and increased violence during the COVID-19 pandemic in South Korea
Source: Front Psychol. 2023 Jul 4;14:1152693. doi: 10.3389/fpsyg.2023.1152693 (PMC10352582; doi:10.3389/fpsyg.2023.1152693)
Supplement: Supplementary file 1 [file Data_Sheet_1.docx]

Supplementary Table 1. Social distancing plans during the COVID-19 pandemic in South Korea

| Duration (M/D/Y) | Terminology | Description |
| --- | --- | --- |
| 03/22/20 – 04/07/20 | Enhanced distancing | Effort to control population density in multi-person facilities |
| 04/08/20 – 04/19/20 | Prohibition of gathering in Seoul | Order to ban gatherings in pubs, bars, and karaoke clubs |
| 04/20/20 – 05/05/20 | Easing some measures | New recommendation from closure to limited operation |
| 05/06/20 – 06/27/20 | Distancing in daily life | Reconciliation of daily life and quarantine |
| 06/28/20 – 11/06/20 | Social distancing (3 levels) | Newly termed social distancing. Introduced level 2.5 on August 5 |
| 11/07/20 – 07/11/21 | Social distancing (5 levels) | Disparate categorization into three areas (metropolitan, non-metropolitan, and Gangwon/JeJu) |
| 07/01/21 – 10/31/21 | Social distancing (4 levels) | Shift from 5 to 4 levels. Level 4: metropolitan area (Seoul, Incheon, and Gyeonggi) |

Supplementary Table 2A. School operation plan during the COVID-19 pandemic in the first semester of 2020

| Grade  Date (M/D) | Elementary | | | | | | Middle | | | High | | |
| --- | --- | --- | --- | --- | --- | --- | --- | --- | --- | --- | --- | --- |
|  | 1 | 2 | 3 | 4 | 5 | 6 | 7 | 8 | 9 | 10 | 11 | 12 |
| 3/2 | Delayed school start | | | | | | | | | | | |
| 4/9 |  | | | | | | | | Online |  | | Online |
| 4/16 |  | | | Online | Online | Online | Online | Online |  | Online | Online |  |
| 4/20 | Online | Online | Online |  |  |  |  |  |  |  |  |  |
| 5/20 |  |  |  |  |  |  |  |  |  |  |  | In-person |
| 5/27 | In-person | In-person |  |  |  |  |  |  | In-person |  | In-person |  |
| 6/3 |  |  | In-person | In-person |  |  |  | In-person |  | In-person |  |  |
| 6/8 |  |  |  |  | In-person | In-person | In-person |  |  |  |  |  |

Supplementary Table 2B. The separated school operation plans during the COVID-19 pandemic in the second semester of 2020

| Metropolitan area (Seoul, Incheon, and Gyeonggi) | | | | Non-metropolitan area | | | |
| --- | --- | --- | --- | --- | --- | --- | --- |
| Date (M/D) | Elementary | Middle | High | Date (M/D) | Elementary | Middle | High |
| 8/16 | 1/3 | 1/3 | 2/3 | 8/24 | 2/3 | 2/3 | 2/3 |
| 8/30 | Stop attending school in person for grade 1-11 | | | 8/26 | 1/3 | 1/3 | 2/3 |
| 9/21 | 1/3 | 1/3 | 2/3 | 9/14 | 1/2 | 1/2 | 2/3 |
| 10/12 | 2/3 | 2/3 | 2/3 | 10/12 | 2/3 | 2/3 | 2/3 |
|  |  |  |  | 10/19 | 1/1* | 1/1* | 1/1* |
| 11/2 | 1/1 | 1/1 | 1/1 | 11/2 | 1/1 | 1/1 | 1/1 |
| 11/19 | 2/3 | 2/3 | 2/3 | 11/24 | 2/3** | 2/3** | 2/3** |
| 12/8 | 1/3 |  | | 12/1 | 2/3 | 2/3 | 2/3 |
| 12/15 | Stop attending school in person | | | 12/23 | Stop attending school in person *** | | |

Note) The numbers in the table indicate the ratio of students who can attend school in person. For instance, 1/3 indicates that only one-third of students can attend school in person.

*Excluding overcrowded schools

**Only for Jeonnam, Jeonbuk, and Gwangju regions

***Only for Daegu and Gyeongbuk regions
